# How do pregnant women’s perceptions of obstetric forceps change according to their demographic background: a cross sectional study

**DOI:** 10.1186/s12884-021-03854-x

**Published:** 2021-05-11

**Authors:** Jasmine M. Hitt, Angela S. Martin, Jordan E. Dietrich, Natasha Ahmed, Gene T. Lee

**Affiliations:** Department of OBGYN, The University of Kansas Health System, 3901 Rainbow Blvd, Kansas City, Kansas 66160 USA

**Keywords:** Attitudes, Obstetric forceps, Operative vaginal delivery, Pregnancy

## Abstract

**Background:**

Women’s attitudes towards obstetric forceps likely contribute to declining use and opportunities for residency training, but formal documentation of women’s attitudes towards obstetric forceps is currently limited. A clearer understanding should help guide our attempts to preserve its use in modern obstetrics and to improve residency training. Our objective is to document women’s attitudes towards obstetric forceps and the influence basic demographic variables have on those attitudes.

**Methods:**

A cross sectional study was performed. We developed a one-time anonymous structured 5-question survey that was given to all women with low-risk pregnancies presenting to our medical center for prenatal care between October 2018–December 2018. The questionnaire asked for the woman’s self-reported age, race, education level and insurance type. The five questions were as follows: (1) Do you think forceps should be used to deliver babies, (2) Is forceps safe for the baby, (3) Is forceps safe for the mother, (4) Do you think forceps can help to lower the cesarean section rate, (5) Do you think physicians in training should learn to place forceps on a real patient. We calculated means and proportions for the responses according to the overall group and various subgroups. Statistical analysis included Kruskall-Wallis or Mann-Whitney tests as appropriate. Results were also adjusted by regression using a Generalized Linear Model. Power calculation showed sample size of 384 was required.

**Results:**

A total of 499 women returned the questionnaire. Response rate was 56.8% (499/878). The findings suggest that women’s perceptions towards forceps are generally negative. Women with white ethnicity, college education or higher and private insurance did have more favorable views than their counterparts, but the majority still had unfavorable views. Age was not shown to have a significant effect on maternal attitude.

**Conclusion:**

Women’s views towards forceps use in the University of Kansas Medical Center are negative and may be contributing to the decline of its use. Improving women’s perceptions of forceps would require multiple different strategies rather than a single focused easily-implemented message. If forceps training continues, such training will rely on a minority of women who will accept forceps use in childbirth.

**Supplementary Information:**

The online version contains supplementary material available at 10.1186/s12884-021-03854-x.

## Background

Operative vaginal births include both vacuums and forceps, but over the past decade forceps skills have become increasingly less preferred [1, 2]. As the number of skilled practitioners and the ability to train residents adequately becomes rare, we come across the question of whether we can do anything to reverse this trend. The risk-averse nature of our profession, the medical-legal climate, women’s preferences for cesarean birth, and reduced opportunities for training in operative vaginal birth overall means that forceps delivery may become a thing of the past in today’s modern obstetric practice.

Residency programs in obstetrics and gynecology (OBGYN) have tried to combat the decline in forceps training with several efforts. Current strategies have included simulation training, increasing the availability of skilled instructors and prioritizing forceps training before vacuum training [3, 4], While simulation training has helped with training efforts, fidelity of these models is not enough to build confidence for real-world clinical practice. Latter efforts have improved training opportunities, but they are still less applicable to hospitals with low operative birth volumes.

A prior editorial accurately suggested that the major issue surrounding forceps training is not the instructor’s intent, but the public’s reluctance over the use of forceps [2]. If the “optics” surrounding forceps delivery are unfavorable, then both doctors and women will prefer to avoid them. If they perceived forceps deliveries more favorable, it could broaden training opportunities for residents. This could limit decline of this skills set, as residents who gain competency during their training are more likely to continue using them throughout their career [5].

Interestingly, while it is assumed that public perceptions of forceps are generally negative, actual documentation of pregnant women’s attitudes/feelings towards forceps is limited in medical literature. There are studies which have looked at women’s postpartum experiences after operative vaginal birth, but antepartum opinions are less documented [6].

The aim of this study is to query pregnant women’s attitudes and identify sociodemographic variables which may influence their views about obstetric forceps in our medical center. We believe that the results of this study will contribute towards identifying strategies for improving women’s perceptions about forceps deliveries, with the end goal of improving resident training and proficiency.

## Methods

This was a cross-sectional study. We included women receiving prenatal care in the low risk prenatal clinic at the University of Kansas Health System who were eligible for vaginal birth. Those who were unable to read English were excluded. There were no specific requirements for gestational age range at the time of survey. A one-time anonymous questionnaire on paper format was given to women between October 2018 and December 2018. The questionnaire was developed for this study. It is uploaded as a supplemental file (“Forceps Survey”). The surveys were handed to women at the time of check-in to their prenatal appointment. They completed the survey in the privacy of their clinic room without any help or instruction from study coordinators or their clinical provider. Surveys were then collected and placed in a confidential folder by medical assistants who were cleaning the room for the next woman. The institutional review board of the University of Kansas considered this study exempt from IRB approval as it is a quality improvement project for the residency program.

We surveyed PubMed for a prior study with a validated survey regarding pregnant women’s attitudes towards forceps, but we did not encounter any. We developed a questionnaire which asked for the respondent’s self-reported age, ethnicity (White, Hispanic, African American or other), education (did not finish high school, high school, college, masters or doctorate) and insurance type (self, private or government). These were considered the common demographic variables for this project. The questionnaire included five questions which were as follows: (1) Do you think forceps should be used to deliver babies? (2) Is forceps safe for the baby? (3) Is forceps safe for the mother? (4) Do you think forceps can help to lower the cesarean section rate? (5) Do you think physicians in training should learn to place forceps on a real patient? For questions 1, 4 and 5 (Q1, 4, 5) the responses were yes (1) or no (0). A Likert scale was provided for questions 2 and 3 (Q2, 3) with the responses ranging from never (1) to always (5). (Supplementary file “Forceps Survey”).

Survey data were analyzed by calculating the mean response for each question and then comparing those means. Favorable responses were considered 4 or 5 on the Likert scale and 1 on the yes/no questions. Unfavorable responses were considered 1 or 2 on the Likert scale and 0 on the yes/no questions. A neutral response was considered 3 on the Likert scale. Data were then subgrouped by common demographic variables such as age, ethnicity, education and insurance status for further analysis. Overall group comparisons were analyzed by Kruskall-Wallis tests, then pairwise comparisons were conducted using Mann-Whitney tests. Since multiple pairwise tests were performed, Bonferroni correction was applied to adjust the significance values. To adjust for confounding, General Linear Model regression was performed. Ethnicity, age, education and insurance status were included as independent variables in the model. Responses to the five survey questions were alternately used as dependent variables. All statistical tests were performed using SPSS software (IBM).

To determine sample size, we assumed that approximately 50% of the population would have a favorable attitude towards forceps. We set our confidence limit for 95%. Required sample size was 384.

## Results

A total of 878 surveys were distributed and 499 women (56.8%) returned the paper questionnaire. Demographic characteristics of the respondents can be seen in Table 1. The women who completed the survey ranged from 17 to 44 years with 315 (63.1%) being white. Insurance and education levels were reflective of the general population within a tertiary hospital. Of the 499 responding women, the percentage answering question one was 89% (*n* = 444/499), question two 94% (*n* = 468/499), question three 93% (*n* = 466/499), question four 86% (*n* = 430/499) and question five 90% (*n* = 449/499).

**Table 1:** Demographics

Demographics	Subgroup	Women 499 (100%)
Age	< 20	13 (2.6%)
	20–29	242 (48.5%)
	30–39	233 (46.7%)
	> 40	11 (2.2%)
Ethnicity	White	315 (63.1%)
	Hispanic	54 (10.8%)
	African American	95 (19%)
	Other	30 (6%)
	Missing	5 (1%)
Education	No High School	21 (4.2%)
	High School	163 (32.7%)
	College	199 (39.9%)
	Masters	75 (15%)
	Doctors	27 (5.4%)
	Missing	14 (2.8%)
Insurance	Self	51 (10.2%)
	Private	285 (57.1%)
	Government	141 (28.3%)
	Missing	22 (4.4%)

In general, respondents had an unfavorable view of forceps (Table 2). Less than half answered that forceps should be used to deliver babies (*n* = 178/444; 40.4%). Most respondents felt that forceps was unsafe for the baby (Q2 score mean 2.48 ± 0.87) and the mother (Q3 score mean 2.65 ± 0.90). Half of the respondents believed that the use of forceps could help reduce the Cesarean section rate (*n* = 215/430; 50%). Slightly more than half did feel that physicians in training could learn forceps on real world women during labor (*n* = 247/449; 55%).

**Table 2:** Subgroup Results by Ethnicity, Age, Education, and Insurance

Question	Overall	Subgroup A	Subgroup B	Subgroup C	Subgroup D
Ethnicity	**All (*n*** **= 499)**	**White(*n*** **= 315)**	**Hispanic(n = 54)**	**African American(*n*** **= 95)**	**Other(*n*** **= 30)**
Q1	40% (n = 444)	49% (*n* = 278)	35% (n = 54)	25% (*n* = 84) ^a^	17% (*n* = 24) ^a^
Q2	2.48 ± 0.87 (*n* = 468)	2.6 ± 0.84 (*n* = 298)	2.26 ± 0.91 (*n* = 54) ^a^	2.31 ± 0.90 (*n* = 86)	2.28 ± 0.94 (n = 25)
Q3	2.65 ± 0.90 (*n* = 466)	2.8 ± 0.84 (*n* = 298)	2.53 ± 0.95 (*n* = 53)	2.36 ± 0.94 (*n* = 85) ^a^	2.76 ± 1.01 (*n* = 25)
Q4	50% (*n* = 430)	56% (*n* = 271)	42% (*n* = 50)	36% (*n* = 81) ^a^	42% (*n* = 24)
Q5	55% (n = 449)	60% (*n* = 287)	37% (*n* = 52) ^a^	53% (*n* = 83)	48% (n = 23)
Age	**All (n = 499)**	**< 20 (n = 13)**	**20–29 (*n*** **= 242)**	**30–39 (*n*** **= 233)**	**> 40 (*n*** **= 11)**
Q1	40% (n = 444)	23% (n = 13)	41% (*n* = 222)	41% (*n* = 202)	43% (*n* = 7)
Q2	2.48 ± 0.87 (n = 468)	2.15 ± 1.34 (n = 13)	2.53 ± 0.87 (*n* = 231)	2.45 ± 0.85 (n = 214)	2.5 ± 0.53 (n = 10)
Q3	2.65 ± 0.90 (*n* = 466)	2.00 ± 1.29 (*n* = 13)	2.65 ± 0.91 (*n* = 229)	2.71 ± 0.85 (*n* = 214)	2.4 ± 0.70 (*n* = 10)
Q4	50% (*n* = 430)	33% (*n* = 12)	51% (*n* = 219)	50% (*n* = 193)	50% (*n* = 6)
Q5	55% (n = 449)	25% (n = 12)	56% (*n* = 225)	57% (*n* = 205)	29% (n = 7)
Education	**All (n = 499)**	**Below High School(n = 21)**	**High School(n = 152)**	**College(n = 190)**	**Post-College(n = 94)**
Q1	40% (n = 444)	29% (n = 21)	36% (*n* = 147)	44% (*n* = 179)	47% (n = 86)
Q2	2.48 ± 0.87 (n = 468)	2.29 ± 1.19 (n = 21)	2.33 ± 0.83 (n = 152)	2.59 ± 0.82 (n = 190)	2.57 ± 0.93 (*n* = 94)
Q3	2.65 ± 0.90 (n = 466)	2.32 ± 1.16 (*n* = 19)	2.53 ± 0.92 (*n* = 152)	2.73 ± 0.81 (*n* = 190)	2.83 ± 0.91 (n = 94)
Q4	50% (n = 430)	33% (*n* = 18)	41% (*n* = 139) ^d^	53% (*n* = 176)	62% (*n* = 87)
Q5	55% (n = 449)	21% (n = 19) ^c, d^	51% (*n* = 144)	60% (*n* = 182)	61% (*n* = 93)
Insurance	**All (n = 499)**	**Private (*n*** **= 285)**	**Government(*n*** **= 141)**	**Self (*n*** **= 51)**	**N/A**
Q1	40% (n = 444)	42% (*n* = 255)	31% (*n* = 131)	58% (n = 43) ^b^	N/A
Q2	2.48 ± 0.87 (n = 468)	2.6 ± 0.87 (*n* = 268)	2.22 ± 0.82 (*n* = 136) ^a^	2.6 ± 0.89 (n = 48) ^b^	N/A
Q3	2.65 ± 0.90 (n = 466)	2.76 ± 0.87 (*n* = 268)	2.40 ± 0.93 (*n* = 134) ^a^	2.81 ± 0.89 (*n* = 48) ^b^	N/A
Q4	50% (*n* = 430)	57% (*n* = 244)	35% (*n* = 128) ^a^	60% (n = 43) ^b^	N/A
Q5	55% (n = 449)	58% (*n* = 257)	52% (*n* = 133)	59% (n = 44)	N/A

Q1, Q4, and Q5 are yes/no questions with 0 = no and 1 = yes. Q2 and Q3 are a 5-point Likert Scale question with 1 = most unfavorable and 5 = most favorable. Subgroup analysis was completed using a Kruskall-Wallis test

^a^ pairwise significance vs Subgroup A with Mann Whitney Test and Bonferroni correction

^b^ pairwise significance vs Subgroup B with Mann Whitney Test and Bonferroni correction

^c^ pairwise significance vs Subgroup C with Mann Whitney Test and Bonferroni correction

^d^ pairwise significance vs Subgroup D with Mann Whitney Test and Bonferroni correction

Subgroup analyses are reported in Table 2. Women of white ethnicity had the most favorable responses, but even they still showed an overall negative attitude towards forceps. Some notable differences were that respondents of other ethnicity had the lowest score on Q1 (17%), indicating that the largest majority believed that forceps should not be used to deliver babies. African-American respondents had the lowest score on Q4 (36%), believing that forceps could not lower the Cesarean rate. Hispanic respondents had the lowest score on Q5 (37%), believing that trainees should not be learning on real world women during labor.

Age < 20 years showed the least favorable attitudes towards forceps. Age > 39 years mostly appeared similar to the other age groups, but their response to Q5 regarding physicians in training learning on real world women during labor was much closer to the youngest age group (Q5 age < 20 score 25% vs Q5 age > 39 score 29%). College graduates made up the largest subgroup by education (190; 39.9%). Those without a high school diploma (21; 4.2%) showed the lowest score on every question and statistical significance was especially profound on Q5 (21% vs college 60% and post-college 61%). High school graduates showed a lower score on Q4 compared to post-college graduates (41% vs 62%). With education level advancing, attitudes towards forceps became increasingly favorable. However, even a majority with graduate degrees still did not feel that forceps should be used for birth (Q1 no response 53%). Both women with college and graduate degrees did feel that resident physicians could train on real world women during labor (Q5 score ~ 60%). The Government insured group (Medicare or Medicaid) had the least favorable responses towards forceps (Q1 no response 69%). This group had statistically significant differences compared to Private insurance in Q2,Q3,Q4 and significant differences compared to Self-pay in Q1,Q2,Q3,Q4, with Private insurance and Self-pay being more favorable. The self-insured group curiously had the most favorable attitude towards forceps, believing they could be used for birth (Q1 yes response 58%), could reduce the cesarean rate (Q4 yes response 60%) and that trainees could learn the skill on real world women during labor (Q5 yes response 59%).

Adjustment of the results was performed to investigate which findings were most robust. The largest sub-groups were considered the reference group for comparisons (Table 3). African-American women and other ethnicities showed stronger negative responses to Q1 compared to their White-American counterparts. Women whose ages were 30–39 years were much less likely than their peers 20–29 years to believe that forceps deliveries could reduce the overall cesarean birth rate (Q4). Individuals without a high school degree showed stronger negative responses to Q5 compared to their college educated counterparts. Self-insured individuals had much more positive responses to Q1 compared to Private insurance individuals and government insured individuals showed lower scores to Q2 compared to Private insurance individuals.

**Table 3:** Adjusted ORs and Differences with 95% Confidence Intervals by Ethnicity, Age, Education, and Insurance

Question	Subgroup A ref	Subgroup B aOR (95% CI)	Subgroup C aOR (95% CI	Subgroup D
Ethnicity	**White**	**Hispanic**	**African American**	**Other**
Q1 (*n* = 416)	OR = 1	0.64 (0.3, 1.3)	0.35 (0.2, 0.7) ^a^	0.16 (0.05, 0.6) ^a^
Q2 (*n* = 438)	Adj diff = 0	−0.23 (− 0.5, 0.04)	− 0.14 (− 0.4, 0.1)	−0.24 (− 0.6, 0.1)
Q3 (*n* = 436)	Adj diff = 0	− 0.07 (− 0.4, 0.2)	−0.2 (− 0.4, 0.04)	0.15 (− 0.2, 0.5)
Q4 (*n* = 403)	OR = 1	0.72 (0.4, 1.4)	0.63 (0.3, 1.2)	0.57 (0.2, 1.4)
Q5 (*n* = 421)	OR = 1	0.51 (0.3, 1.01)	1.2 (0.7, 2.2)	0.86 (0.3, 2.2)
Age	**20–29**	**< 20**	**30–39**	**> 40**
Q1 (n = 416)	OR = 1	0.69 (0.2, 2.9)	0.87 (0.6, 1.4)	0.9 (0.1, 5.5)
Q2 (n = 438)	Adj diff = 0	−0.14 (−0.6, 0.4)	−0.17 (− 0.3, 0.1)	0.2 (− 0.4, 0.8)
Q3 (n = 436)	Adj diff = 0	−0.44 (− 0.9, 0.1)	−0.03 (− 0.2, 0.1)	0.01 (− 0.6, 0.6)
Q4 (n = 403)	OR = 1	0.83 (0.2, 3.1)	0.62 (0.4, 0.9) ^a^	0.75 (0.1, 5)
Q5 (n = 421)	OR = 1	0.26 (0.06, 1.03)	0.82 (0.5, 1.3)	0.27 (0.05, 1.6)
Education	**College**	**Below High School**	**High School**	**Post-College**
Q1 (n = 416)	OR = 1	0.87 (0.3, 2.8)	0.85 (0.5, 1.5)	1.2 (0.7, 2.1)
Q2 (n = 438)	Adj diff = 0	−0.01 (−0.4, 0.4)	−0.13 (− 0.3, 0.1)	−0.05 (− 0.3, 0.2)
Q3 (n = 436)	Adj diff = 0	−0.23 (− 0.7, 0.2)	−0.04 (− 0.3, 0.2)	0.06 (− 0.2, 0.3)
Q4 (n = 403)	OR = 1	0.7 (0.2, 2.2)	0.75 (0.4, 1.3)	1.49 (0.9, 2.6)
Q5 (n = 421)	OR = 1	0.2 (0.1, 0.7) ^a^	0.66 (0.4, 1.2)	1.13 (0.7, 1.9)
Insurance	**Private**	**Government**	**Self**	**N/A**
Q1 (n = 416)	OR = 1	1.05 (0.6, 2)	2.7 (1.3, 5.6) ^a^	N/A
Q2 (n = 438)	Adj diff = 0	−0.3 (−0.5, −0.1) ^a^	0.07 (−0.2, 0.3)	N/A
Q3 (n = 436)	Adj diff = 0	−0.21 (− 0.5, 0.04)	0.12 (− 0.2, 0.4)	N/A
Q4 (n = 403)	OR = 1	0.6 (0.3, 1.1)	1.55 (0.8, 3.2)	N/A
Q5 (n = 421)	OR = 1	1.28 (0.7, 2.3)	1.34 (0.7, 2.7)	N/A

^a^ significant vs Subgroup A using a General Linear Model regression

## Discussion

This study suggests that women’s perceptions towards forceps are generally negative. Women of all demographics were more likely to believe that forceps should not be used because they are unsafe for mother and baby. In addition, many did not believe forceps could lower the cesarean birth rate. Consistent with overall perceptions, about half believed that resident physicians could be allowed to learn to place forceps on a live volunteer. White ethnicity and higher education levels tended to have more favorable attitudes, while extremes of age or government insurance status indicated more unfavorable attitudes.

These attitudes were not surprising and could be explained by several possibilities. One simple reason is that women are simply reflecting similar ambivalences apparent within obstetrical providers in current practice. When used incorrectly, forceps can clearly lead to neonatal and maternal trauma. As documented elsewhere, rates of forceps deliveries have decreased compared to vacuum deliveries [1, 2]. Concern about litigation has been documented, but decreased use also appears to be partly due to practitioner’s preference [7]. Sometimes this preference reflects decreased training opportunities. Some residency programs only choose to teach vacuum deliveries rather than forceps. In other instances, providers may have trained in residency, but have since abandoned the practice. Another reason for the unfavorable attitudes towards forceps could be a recognized trend in women’s and providers’ increasingly favorable attitudes for cesarean birth overall [8]. While most women aim for spontaneous vaginal birth, there is a clear minority of women who would choose elective cesarean outright [9, 10]. In general, cesarean births appear to be desired by both women and providers rather than engage in difficult operative vaginal births, whether vacuum or forceps. There are even current reports which wonder whether training for vacuum operative births will also become scarce [11]. Both of these trends may mean that most women feel that operative vaginal births are the lesser option compared to cesarean. Unfortunately, this survey did not compare those two options directly, but it would be an interesting follow up study.

African Americans did show less favorable attitudes towards forceps and these are worth exploring briefly. African Americans consistently show poorer outcomes in pregnancy compared to their White-American counterparts [12–14]. They also experience bias and prejudice in the medical system [15, 16]. Historical cases such as the Tuskegee syphilis experiment have created reasons for distrust of medical professionals within the African American community [17]. These reasons may all be working to create even more unfavorable impressions towards obstetric forceps, a tool which already comes with significant debate about its use. Hispanics and other ethnicities may have shown unfavorable attitudes due to larger representation of immigrants within those groups. For example, immigrants in the Netherlands reported issues of communication, autonomy and respect [18]. An Australian sponsored study looked at five host countries with significant immigrant populations and also found issues with communication and discrimination [19]. In the United States, anti-immigrant bias is no less common and can be seen in Hispanic, Asian and other ethnicities [20]. These examples of problematic communication likely contribute to distrust of medical professionals or the health care system by these groups [17, 21].

Our analysis of educational background suggested that only those without a high school diploma were especially skeptical of forceps. This is likely due to lower health literacy. Lower levels of education are highly predictive of low health literacy [22]. In turn, prior studies have shown that lower health literacy is linked to lower levels of trust in physicians [23, 24].

Prior studies showed that women with Medicare or Medicaid have lower health literacy than those with private insurance [25, 26]. Similarly, studies of self-insured women have also shown lower health literacy and a tendency to avoid participating in clinical trials or research due to safety concerns [26–28]. In this study, however, women with self-insurance had more favorable views of forceps (Q1, 58% favorable) while government-insured ones had more unfavorable views of forceps (Q1, 31% favorable). Both subgroups had similar ethnicity and education levels. Lower levels of health literacy about forceps would be consistent with prior survey observations as an explanation to why government-insured women had more unfavorable attitudes, but it would not explain the self-insured ones’ attitudes. This subgroup may require further study as our sample size of self-insured women was small.

Age surprisingly did not produce statistical differences. Teenagers would be expected to have the lowest health literacy and they showed the least favorable attitudes towards forceps. Low numbers of teenagers, however, may have played a role in the absence of any statistical finding. We did find a statistically lower number of women aged 30–39 who believed that forceps could reduce the cesarean birth rate but this was isolated and the other age categories did not support a trend. It is worth noting that age > 39 produced more unfavorable attitudes similarly as teenagers towards trainees practicing on real world women during labor in our study. Many women with advanced maternal age consider themselves “high risk” and may seek to reduce any risk to their pregnancy outcomes [29]. This could easily exclude allowing trainees to participate in their care. More study is needed on the effect of age on attitudes towards medical trainees’ education.

There are several strengths to this study. To our knowledge, there have been no studies that document women’s perceptions towards forceps. The demographic characteristics of the women who completed the survey are applicable to university settings. There were enough respondents to confidently determine whether differences existed. Additionally, the survey layout was simple and easy to understand, therefore most women had given answers to every question.

This study is also subject to several limitations. First, we did not include clinical history such as parity or prior operative birth in our survey, and we would expect that women’s personal experiences would be extremely important regarding their perspectives of forceps use. In fact, such clinical history might well override the demographic associations we observed in our study. Our intention was to provide an initial look at how attitudes might segregate based on simple demographic variables, and we welcome further study into how prior clinical experiences might modify those attitudes. Second, our results might not be generalizable outside university settings. Community training programs may have different sets of assumptions governing clinical care and training protocols. Lastly, survey data are subject to responders’ bias, which may skew results.

## Conclusion

Women’s attitudes towards forceps in the University of Kansas Medical Center are generally negative and affected by several demographic characteristics. Improving women’s perceptions of forceps would require multiple different strategies rather than a single focused easily-implemented message. More research is needed to determine if this negativity exists in other academic hospitals as well as whether a particular subset of women exists who would be most receptive to the idea of forceps continuing to be used in modern obstetric practice. It seems likely that if forceps training continues, such training will rely on a minority of women who will accept forceps use in childbirth.

## Supplementary Information

**Additional file 1.** Maternal Attitudes About Forceps Survey, survey form filled out by the study participants.

## Data Availability

The datasets used and/or analyzed during the current study are available from the corresponding author on reasonable request.

## Abbreviations

OBGYN: Obstetrics and gynecology
Q1–5: question 1–5
