# Supplementary material for: How do pregnant women’s perceptions of obstetric forceps change according to their demographic background: a cross sectional study
Source: BMC Pregnancy Childbirth. 2021 May 11;21:371. doi: 10.1186/s12884-021-03854-x (PMC8111760; doi:10.1186/s12884-021-03854-x)
Supplement: Supplementary file 1 — Additional file 1. Maternal Attitudes About Forceps Survey, survey form filled out by the study participants. [file 12884_2021_3854_MOESM1_ESM.pdf]

## MATERNAL ATTITUDES ABOUT FORCEPS SURVEY

1. Age _________

2. Ethnicity

| | | | |
|---|---|---|---|
| 1 white | 2 Hispanic | 3 African-American | 4 Other |

3. Highest level of education completed __________________

4. Insurance level

| | | |
|---|---|---|
| 1 self-insured | 2 private insurance | 3 Medicaid/public |

Attitude –

1. Do you think forceps should be used to deliver babies?

| | |
|---|---|
| YES | NO |

2. Is forceps safe for the baby?

| | | | | |
|---|---|---|---|---|
| 1 Never | 2 Sometimes | 3 Neutral | 4 Mostly | 5 Always |

3. Is forceps safe for the mother?

| | | | | |
|---|---|---|---|---|
| 1 Never | 2 Sometimes | 3 Neutral | 4 Mostly | 5 Always |

4. Do you think forceps can help to lower the cesarean section rate?

| | |
|---|---|
| YES | NO |

5. Do you think training physicians should learn to place forceps on a real patient?

| | |
|---|---|
| YES | NO |
